# Vericiguat in the Post-Stabilization Phase of HFrEF: Targeting Residual Risk Across the Ischemia–Decompensation Continuum

**DOI:** 10.3390/ijms27125301

**Published:** 2026-06-11

**Authors:** Beata Krasińska, Calogera Pisano, Roberta Vazzana, Giuseppe Maria Raffa, Mariusz Kowalewski, Krzysztof J. Filipiak, Jarosław Bartkowski, Zbigniew Krasiński, Piotr Suwalski, Kinga Koziarska, Vincenzo Nuzzi, Paolo Manca, Gennaro Galasso, Tomasz Urbanowicz

**Affiliations:** 1 Department of Hypertensiology, Angiology, and Internal Medicine, Poznan University of Medical Sciences, ½ Długa Street, 61-848 Poznań, Poland; 2 Department of Research, IRCCS-ISMETT (Mediterranean Institute for Transplantation and Specialized Therapies), 90127 Palermo, Italy; rvazzana@ismett.edu (R.V.);; 3 Department of Precision Medicine in Medical Surgical and Critical Area (Me.Pre.C.C.), University of Palermo, 90134 Palermo, Italy; 4 Department of Cardiac Surgery, Center of Postgraduate Medical Education, Central Clinical Hospital of the Ministry of Interior, 02-507 Warszawa, Poland; 5 The Centre of Postgraduate Medical Education, 99/103 Marymoncka Street, 01-813 Warsaw, Poland; 6 Department of Cardiac Surgery and Transplantology, Poznan University of Medical Sciences, ½ Długa Street, 61-848 Poznan, Poland; 7 Department of Vascular, Endovascular Surgery, Angiology and Phlebology, Poznan University of Medical Science, ½ Dluga Street, 61-848 Poznan, Poland; 8 University Clinical Hospital, 49 Przybyszewskiego Street, 60-355 Poznan, Poland; 9 Department of Clinical Cardiology and Heart Failure, IRCCS ISMETT (Mediterranean Institute for Transplantation and Advanced Specialized Therapies), 90127 Palermo, Italy; 10 Department of Medicine, Surgery, and Dentistry, University of Salerno, 84084 Salerno, Italy

**Keywords:** HF, vericiguat, ACS, AHF

## Abstract

Vericiguat is currently indicated for patients with heart failure with reduced ejection fraction (HFrEF) following recent clinical worsening, based on evidence demonstrating a reduction in cardiovascular death or heart failure hospitalization in a high-risk population. While this positioning is clinically justified, it may underestimate the broader pathophysiological context in which soluble guanylate cyclase (sGC) stimulation may be relevant, particularly in phases of persistent biological activation following apparent clinical stabilization. In routine practice, acute coronary syndromes (ACS), acute heart failure (AHF), and chronic HFrEF are approached as distinct clinical entities. However, these conditions often represent sequential manifestations of a continuous disease trajectory driven by persistent endothelial dysfunction, impaired nitric oxide–sGC–cyclic guanosine monophosphate (NO–sGC–cGMP) signaling, and residual vascular risk. In this perspective, we revisit the mechanistic and clinical rationale for vericiguat and propose a reframing of its therapeutic role. Its greatest utility may lie in patients with recently worsening HFrEF who remain biologically vulnerable after stabilization. Extension of this concept to post-ACS populations remains hypothesis-generating and is not supported by direct clinical evidence. This “post-stabilization vulnerable state” represents a clinically recognizable yet insufficiently targeted phase, characterized by ongoing biological activation despite apparent clinical improvement. Adopting a continuum-based view of cardiovascular disease may improve alignment between pathophysiology and treatment, refine patient selection, and inform future trial design focused on this early post-event window. Importantly, this perspective is hypothesis-generating and reflects an effort to align emerging mechanistic insights with clinical trajectory, rather than to extend current indications beyond the available evidence base.

## 1. Introduction

Over the past decades, the management of cardiovascular disease has been shaped by the development of robust, disease-specific evidence bases. Acute coronary syndromes (ACS), acute heart failure (AHF), and chronic heart failure with reduced ejection fraction (HFrEF) are each supported by distinct therapeutic algorithms, reflecting differences in clinical presentation, pathophysiology, and trial design [1,2,3,4,5,6,7]. This structured approach has contributed substantially to improved outcomes.

Nevertheless, such compartmentalization may also obscure an important biological reality. In many patients, these syndromes do not occur in isolation but represent different temporal expressions of a shared disease trajectory. An episode of myocardial ischemia may be followed by ventricular dysfunction, neurohormonal activation, and recurrent decompensation, culminating in chronic heart failure. Conversely, patients with established HFrEF may experience recurrent episodes of decompensation, often triggered by ischemia or other stressors.

Despite this continuity, therapeutic strategies are rarely designed to address the transitions between these states. In particular, the period following initial stabilization after an acute event—whether ischaemic or decompensated—remains relatively underexplored from a therapeutic perspective [8,9]. This phase is clinically characterized by apparent stabilization, yet biologically it remains highly active, with ongoing neurohormonal, inflammatory, and endothelial perturbation that may not be adequately addressed by current therapies.

Vericiguat, an oral stimulator of soluble guanylate cyclase (sGC), has been introduced into clinical practice based on its efficacy in patients with recently worsening HFrEF [10,11]. However, its mechanism of action suggests that its therapeutic relevance may extend beyond this narrowly defined indication. Specifically, it may target a component of residual pathophysiology that persists across different phases of cardiovascular disease.

In this article, we examine the mechanistic basis and clinical evidence for vericiguat and propose that its optimal role may lie within a broader conceptual framework—namely, the ischemia–decompensation continuum. Within this model, we identify a distinct post-stabilization phase in which targeted intervention may be particularly beneficial.

Importantly, the present framework is primarily intended to describe patients with established HFrEF following a recent worsening event. Although acute coronary syndromes may contribute to the development of this vulnerable state in selected patients, the available clinical evidence supporting vericiguat remains confined to worsening HFrEF populations rather than ACS populations per se.

Despite substantial advances in guideline-directed medical therapy, a considerable proportion of patients experience recurrent events shortly after apparent clinical stabilization. This early post-event period is characterized by a paradox: clinical improvement coexists with persistent biological activation and a high residual risk of rehospitalization and mortality. Current therapeutic strategies are largely optimized either for acute instability or long-term disease modification, with comparatively little focus on this transitional phase. As a result, an important window for intervention may remain underexploited.

**Figure 1:**
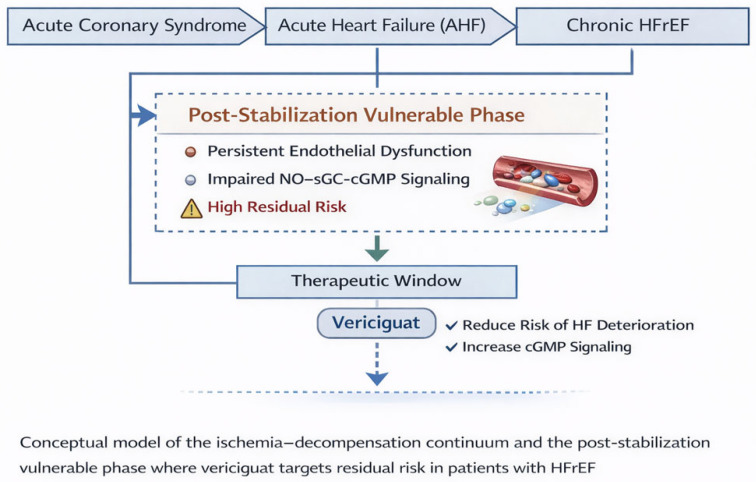
Conceptual model, presenting the variciguat role in the post-stabilization phase in chronic and acute HFrEF related to ACS. Abbreviations: AHF—acute heart failure, cGMP—Cyclic Guanosine MonophosphateHF—heart failure, HFrEF—heart failure with reduced ejection fraction, NO-sGC-cGMP—Nitric Oxide (NO)-soluble guanylate cyclase (sGC)—cyclic guanosine monophosphate (cGMP).

Although the concept of a ‘vulnerable phase’ following heart failure hospitalization has been previously described, it is typically framed in clinical rather than mechanistic terms. In contrast, we propose a biologically anchored definition of this period, characterized by persistent endothelial dysfunction and impaired NO–sGC–cGMP signaling despite apparent hemodynamic recovery (Figure 1).

Throughout this review, a distinction should be made between evidence derived from randomized clinical trials and mechanistic hypotheses based on the biological role of the NO–sGC–cGMP pathway. While vericiguat has demonstrated clinical benefit in worsening HFrEF, its proposed role in broader post-stabilization states remains speculative and requires prospective validation.

## 2. The Ischaemia–Decompensation Continuum

### From Discrete Entities to a Unified Trajectory

The traditional separation of ACS, AHF, and chronic HFrEF reflects differences in clinical presentation and management priorities [12,13]. However, from a pathophysiological perspective, these conditions are closely interrelated.

ACS initiates a cascade of events that includes myocardial injury, inflammation, and endothelial dysfunction [14,15]. Nevertheless, not all patients experiencing ACS progress to worsening HFrEF, and the biological continuum proposed here should not be interpreted as implying a uniform disease trajectory. Rather, ACS should be viewed as one potential contributor to the pathophysiological processes that may culminate in recurrent HF worsening in susceptible individuals. Even after successful revascularisation, many patients exhibit persistent abnormalities in vascular and myocardial function [16,17]. These abnormalities may contribute to adverse remodeling, reduced ejection fraction, and progression to heart failure.

Similarly, episodes of AHF often occur in patients with underlying structural heart disease and are associated with further deterioration in cardiac function [18,19]. Recurrent decompensation is common and contributes to a progressive decline in clinical status [20,21,22].

Taken together, these observations support a model in which cardiovascular disease evolves along a continuum, rather than as a series of discrete events. Within this continuum, different phases can be identified, each characterized by specific pathophysiological features and clinical risks.

## 3. The Post-Stabilisation Vulnerable State

A particularly important phase in heart failure pathophysiology is the period following initial stabilization after an acute event [23]. Clinically, patients may appear improved, with resolution of congestion and haemodynamic stabilisation. However, underlying pathophysiological abnormalities often persist.

This phase may be described as a “post-stabilisation vulnerable state”, characterized by:

- Residual endothelial dysfunction
- Persistent neurohormonal activation
- Ongoing microvascular impairment
- Incomplete reversal of myocardial injury
- Elevated risk of recurrent decompensation

For the purposes of this review, the post-stabilization vulnerable state can be operationally defined as a transitional phase in patients with established HFrEF after a recent worsening event, once acute hemodynamic instability has resolved, but residual biological and clinical risk remains high. Clinically, this state may be recognized in patients with LVEF ≤ 40%, recent hospitalization for HF or intravenous diuretic-treated worsening HF within the previous 3 months, absence of ongoing intravenous vasoactive or diuretic therapy for at least 24 h, and systolic blood pressure ≥ 100 mmHg. Risk enrichment features include persistently elevated NT-proBNP, residual congestion, renal dysfunction, diabetes, ischemic etiology, recent ACS-triggered decompensation, or recurrent HF events.

Epidemiological data indicate that the risk of rehospitalization and death is highest in the weeks and months following an acute event, even after apparent stabilization [24,25]. This suggests that current therapies may not fully address the underlying drivers of risk during this period.

Residual endothelial dysfunction refers to the persistence of impaired endothelial homeostasis despite apparent clinical stabilization. It is characterized by reduced nitric oxide bioavailability, increased oxidative stress, pro-inflammatory activation, impaired vasodilatory capacity, and microvascular dysfunction. These abnormalities may persist for weeks or months following ACS or acute HF episodes and contribute to ongoing cardiovascular risk. In routine clinical practice, direct assessment of endothelial function remains challenging. However, surrogate markers, including elevated NT-proBNP, high-sensitivity C-reactive protein (hsCRP), impaired flow-mediated dilation (FMD), peripheral arterial tonometry, coronary microvascular dysfunction indices, and biomarkers reflecting endothelial activation, may help identify patients with persistent vascular dysfunction. Although these tools are not routinely incorporated into guideline-directed care, they provide a framework for future biomarker-guided therapeutic strategies.

At present, no validated clinical algorithm exists to identify patients for vericiguat based on endothelial dysfunction. Current patient selection remains driven by clinical worsening events and natriuretic peptide levels, whereas endothelial biomarkers should be regarded as investigational.

From a therapeutic standpoint, this phase represents a potential window of opportunity. Interventions targeting residual pathophysiology, rather than acute instability or long-term remodeling alone, may be particularly relevant.

The post-stabilization phase is characterized by multiple pathophysiological derangements described in detail in Table 1.

Importantly, this phase should not be viewed simply as a recovery period, but rather as a distinct therapeutic window in which residual pathophysiology may be more modifiable than in later, chronically stable stages of disease.

## 4. Pathophysiological Rationale: The NO–sGC–cGMP Axis

### 4.1. Central Role in Cardiovascular Homeostasis

The nitric oxide–soluble guanylate cyclase–cyclic guanosine monophosphate (NO–sGC–cGMP) pathway plays a central role in regulating vascular tone, myocardial function, and cellular signaling [31,37]. Nitric oxide (NO), produced by endothelial cells, activates sGC in vascular smooth muscle cells, thereby generating cGMP [26,32]. This, in turn, contributes to vasodilation, modulation of platelet activity, and a range of downstream effects that may include anti-inflammatory and anti-fibrotic signaling.

### 4.2. Dysregulation Across Disease States

In both ischaemic heart disease and heart failure, this pathway is disrupted. Oxidative stress reduces NO bioavailability, while inflammation and endothelial dysfunction further impair signaling [27,29]. As a result, cGMP production is diminished, contributing to:

- Impaired vasodilation
- Increased vascular stiffness
- Microvascular dysfunction
- Adverse myocardial remodeling.

This pathway can be conceptualized as a cascade in which oxidative stress and inflammation reduce endothelial NO bioavailability, leading to impaired sGC activation, diminished cGMP generation, increased vascular stiffness, microvascular dysfunction, and ultimately greater susceptibility to recurrent HF worsening. Vericiguat intervenes downstream of NO by directly stimulating sGC and thereby partially restoring cGMP signaling in a setting where endogenous NO-dependent signaling is impaired.

Importantly, these abnormalities are not confined to advanced disease. They are present during ACS and AHF and often persist despite revascularisation and guideline-directed medical therapy.

### 4.3. Residual Risk and Incomplete Correction

Contemporary therapies for heart failure, including renin–angiotensin system inhibitors, beta-blockers, mineralocorticoid receptor antagonists, and sodium–glucose co-transporter 2 inhibitors, address key neurohormonal pathways and have substantially improved outcomes [28,30]. However, they do not directly target the NO–sGC–cGMP pathway.

As a result, residual dysfunction in this pathway may contribute to ongoing risk, particularly in the post-stabilization phase. This provides a mechanistic rationale for therapies that can restore cGMP signaling independently of NO availability.

### 4.4. Molecular Consequences of Impaired NO–sGC–cGMP Signaling

Beyond its hemodynamic consequences, disruption of the NO–sGC–cGMP pathway exerts broad molecular effects that contribute to disease progression. Reduced intracellular cGMP availability leads to diminished activation of protein kinase G (PKG), a central regulator of vascular homeostasis, myocardial remodeling, calcium handling, and cellular stress responses. Reduced PKG activity promotes endothelial dysfunction, vascular stiffening, and increased susceptibility to inflammatory activation.

At the myocardial level, impaired cGMP–PKG signaling facilitates maladaptive hypertrophic responses, extracellular matrix deposition, and activation of profibrotic pathways, including transforming growth factor-beta (TGF-β) signaling. Experimental studies have further demonstrated that reduced PKG activity contributes to oxidative stress amplification through endothelial nitric oxide synthase uncoupling and increased reactive oxygen species generation.

Emerging evidence also suggests interactions between cGMP signaling and calcium/calmodulin-dependent protein kinase II (CaMKII), a key mediator of electrical instability and arrhythmogenesis. Reduced cGMP signaling may facilitate CaMKII activation, abnormal calcium cycling, and ventricular arrhythmias, whereas restoration of cGMP signaling may attenuate these processes. Collectively, these molecular mechanisms provide biological support for targeting the NO–sGC–cGMP axis beyond simple vasodilation and may partially explain the residual-risk phenotype observed in recently worsening HFrEF.

## 5. Mechanism of Action of Vericiguat

Vericiguat directly stimulates sGC, enhancing cGMP production even in conditions of reduced NO availability [38]. This distinguishes it from nitrates and other therapies that rely on upstream signaling [33,39].

**Figure 2:**
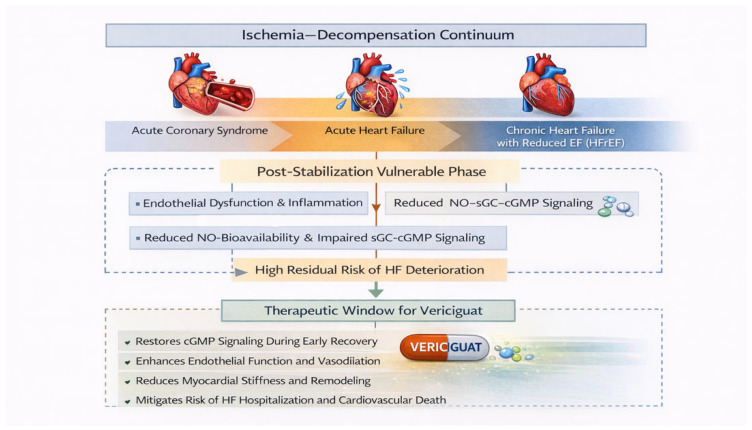
Mechanism of vericiguat action in the post-stabilization vulnerable phase. Abbreviations: cGMP—cyclic guanosine monophosphate, HFrEF—heart failure with reduced ejection fraction, NO—nitric oxide, NO–sGC–cGMP pathway—nitric oxide–soluble guanylate cyclase–cyclic guanosine monophosphate pathway, sGC—soluble guanylate cyclase.

By restoring cGMP levels, vericiguat may contribute to improvements in vascular function and myocardial stress signaling, with potential downstream effects on remodeling, although these mechanisms remain incompletely defined in clinical populations [40]. Importantly, its mechanism is well aligned with the pathophysiological abnormalities observed in the post-stabilization phase. The vericiguat mechanistic properties in the post-stabilization vulnerable phase are presented in Figure 2.

### 5.1. Clinical Evidence

#### VICTORIA: Targeting High-Risk Patients

The VICTORIA trial evaluated vericiguat in patients with HFrEF and recent worsening [35,36], defined by recent hospitalization or the need for intravenous diuretics. The study demonstrated a modest but statistically significant reduction in the composite endpoint of cardiovascular death or heart failure hospitalization.

A notable feature of the trial is the high-risk nature of the population, with elevated natriuretic peptide levels and a high event rate. Patients were enrolled after clinical stabilization, rather than during acute decompensation.

These characteristics suggest that the observed benefit reflects an effect in patients with persistent vulnerability following recent worsening. Following positive results from the VICTORIA trial in patients with a recent worsening event, a randomized, double-blind, placebo-controlled phase III trial, the VICTOR, was conducted at 616 centers in 42 countries. In contrast, studies evaluating vericiguat in more stable HFrEF populations have not demonstrated a comparable magnitude of benefit, underscoring the importance of clinical context and baseline risk. These observations suggest that the efficacy of sGC stimulation may be closely linked to the presence of ongoing pathophysiological activation, rather than stable chronic disease alone. The VICTOR results showed significant secondary endpoint benefits, including a 17% reduction in cardiovascular (CV) mortality, followed by a 25% reduction in all-cause mortality and sudden cardiac death [34]. Concluding, vericiguat did not reduce hospitalizations in stable patients, but it did reduce death, especially sudden death. This suggests that the mortality benefit may be independent of decompensation prevention, possibly through anti-arrhythmic mechanisms (CaMKII inhibition, APD prolongation), as presented in Table 2.

However, these observations should be interpreted cautiously. The mortality and sudden-death signals should be regarded as exploratory and hypothesis-generating, particularly because they derive from secondary or supportive analyses rather than from a trial primarily designed to test anti-arrhythmic mechanisms. Dedicated mechanistic studies and adequately powered prospective trials are required before vericiguat can be positioned as an anti-arrhythmic or sudden-death–modifying therapy.

Notably, the magnitude of benefit observed in VICTORIA was modest and primarily driven by reductions in heart failure hospitalization rather than mortality, which should be taken into account when considering broader application of this therapy.

Within the contemporary therapeutic landscape, vericiguat occupies a distinct mechanistic niche. Unlike neurohormonal antagonists, which primarily modulate maladaptive signaling pathways, or sodium–glucose co-transporter 2 inhibitors, which exert pleiotropic metabolic and hemodynamic effects, vericiguat directly targets impaired cGMP signaling downstream of nitric oxide. This distinction is particularly relevant in the post-stabilization phase, where endothelial dysfunction and reduced NO bioavailability persist despite otherwise optimized therapy. In this context, sGC stimulation may address a component of residual risk that is not adequately targeted by existing drug classes.

### 5.2. Context-Dependent Efficacy

In contrast, studies conducted in more stable populations have not demonstrated a comparable benefit [41,42]. This divergence highlights the importance of patient selection and clinical context.

The available data suggest that vericiguat does not exert uniform effects across the heart failure spectrum, but rather that its benefit is closely dependent on timing, disease trajectory, and the degree of residual biological activation

### 5.3. Emerging Data in Post-ACS Populations

Data specifically evaluating vericiguat in post-ACS populations remain limited and insufficient to support routine clinical use in this setting [43]. Although endothelial dysfunction, impaired NO–sGC–cGMP signaling, and residual cardiovascular risk may persist following ACS, direct evidence demonstrating clinical benefit from sGC stimulation in patients without worsening HFrEF is currently lacking. Therefore, any extension of the post-stabilization concept to post-ACS populations should be considered exploratory and hypothesis-generating.

These findings should be interpreted with caution but are consistent with the proposed role of vericiguat in the post-stabilization phase. Accordingly, extension of this therapeutic concept to post-ACS populations should be considered hypothesis-generating and require prospective validation.

## 6. Clinical Implications

### 6.1. Refining Patient Selection

The available evidence supports a targeted approach to vericiguat use. Patients most likely to benefit include those with:

- HFrEF
- Recent worsening events
- Clinical stabilisation
- Persistent risk despite optimal therapy

This aligns closely with the population studied in VICTORIA but may also extend to selected patients following ACS.

**Figure 3:**
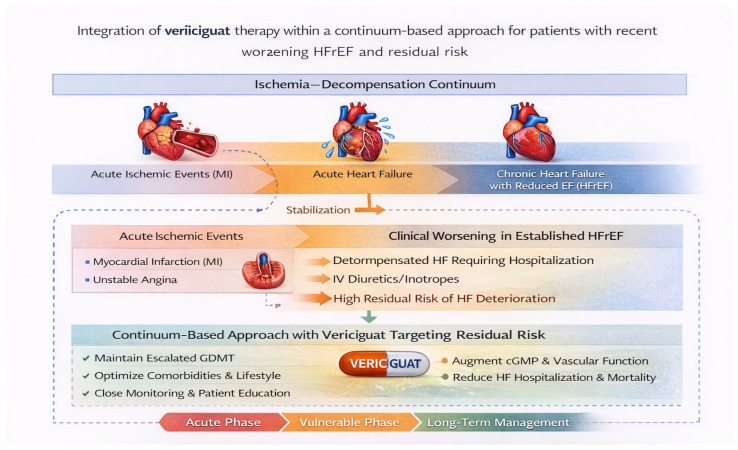
Integration of vericiguat into HFrEF therapy. Abbreviations: cGMP—cyclic guanosine monophosphate, GDMT—guideline-directed medical therapy, HF—heart failure, HFrEF—heart failure with reduced ejection fraction, IV—intravenous, MI—myocardial infarction.

Vericiguat should be considered as complementary to, rather than a replacement for, established therapies. Its role is to address a specific component of residual risk that is not fully targeted by current treatment strategies, as presented in Figure 3.

#### 6.1.1. Current Expert Guidance Identifies Vericiguat Candidates as:

1. Clear indication:
  - HFrEF with recent worsening event (hospitalization or IV diuretics) despite GDMT
2. Also consider when:
  - Intolerant to MRAs (eGFR < 30 or hyperkalemia)
  - Unable to reach ≥50% target doses of foundational therapies
  - Persistent high NT-proBNP despite quadruple therapy
  - History of sudden cardiac death or ventricular arrhythmias (given VICTOR’s SCD reduction)

In practical terms, this translates into considering vericiguat in patients who, despite guideline-directed therapy, remain at high short-term risk as reflected by recent decompensation, persistently elevated natriuretic peptides, and incomplete physiological recovery.

#### 6.1.2. Proposed Target Population:

The post-stabilization vulnerable state discussed in this review primarily refers to patients with established HFrEF who have experienced a recent worsening event, including hospitalization for HF or the need for intravenous diuretics, consistent with the VICTORIA population. We additionally discuss patients whose worsening episode was precipitated by ACS and who subsequently develop persistent LV dysfunction. In contrast, patients with ACS without HF, transient LV dysfunction after MI, or isolated ischemic cardiomyopathy without recent worsening HF represent distinct populations for which evidence supporting vericiguat remains insufficient.

### 6.2. Timing of Initiation

Timing appears to be critical. Initiation during acute instability is not supported by current evidence, while delayed initiation in stable patients may reduce potential benefit.

The available data suggest that the optimal window for initiation lies early after clinical stabilization, when residual pathophysiology remains active but hemodynamic conditions permit the safe introduction of additional therapy.

Based on the VICTORIA trial and clinical guidance, the window can be operationalized, initiating vericiguat once the patient is stabilized after decompensation (≥24 h without IV medications) and SBP ≥ 100 mmHg, as shown in Table 3. The therapy should be started with a 2.5 mg dose and titrated every two weeks to 10 mg/daily.

Patients with worsening HF have up to 10% mortality and 30% rehospitalization within weeks after an AHF episode [18,23,45]. In epidemiological studies, approximately 1 in 4 patients with HFrEF and a recent worsening event either die or have another worsening event within 30 days [44,46,47]. This risk persists despite quadruple therapy (ARNI + BB + MRA + SGLT2i) [48,49,50,51]. Vericiguat addresses the component of residual risk not captured by neurohormonal blockade—specifically, NO-sGC-cGMP pathway dysfunction.

Importantly, HFrEF should not be regarded as a homogeneous entity when considering vericiguat. Patients with ischemic cardiomyopathy, recurrent ACS-triggered decompensation, chronic kidney disease, diabetes, advanced age, frailty, or persistently elevated natriuretic peptides may represent phenotypes with particularly high residual risk. Conversely, clinically stable patients without recent worsening, low natriuretic peptide burden, or transient LV dysfunction after ACS without established HF should not be assumed to derive the same benefit. Future studies should clarify whether ischemic versus non-ischemic etiology, renal dysfunction, metabolic disease, or arrhythmic vulnerability modifies the treatment response.

## 7. Integration into Therapeutic Pathways

Vericiguat should be positioned within the contemporary heart failure therapeutic landscape, not as a competitor to foundational neurohormonal blockade, but as a mechanistically complementary strategy directed at persistent residual risk in patients who remain vulnerable after recent worsening heart failure.

Importantly, the VICTORIA trial was conducted before widespread implementation of contemporary quadruple therapy [52,53,54]. Background use of SGLT2 inhibitors was negligible, and ARNI utilization was substantially lower than current standards. Consequently, the magnitude of benefit observed with vericiguat may not directly translate to contemporary populations receiving optimized quadruple therapy. Future studies are required to define its incremental value when added to ARNI, beta-blockers, MRAs, and SGLT2 inhibitors.

Unlike angiotensin receptor–neprilysin inhibition (ARNI), which primarily amplifies endogenous natriuretic peptide signaling while suppressing maladaptive renin–angiotensin activity, or sodium–glucose cotransporter 2 inhibitors (SGLT2i), which exert broad cardiorenal and metabolic benefits across the heart failure spectrum, vericiguat uniquely targets downstream sGC–cGMP signaling in the setting of impaired nitric oxide bioavailability, addressing a pathway not directly modulated by other foundational therapies.

This niche becomes particularly relevant in the early post-stabilization phase, when congestion may have improved, but biological perturbation, vascular dysfunction, and recurrent event risk remain high. In contrast, omecamtiv mecarbil represents a fundamentally different paradigm centered on selective cardiac myosin activation and systolic performance enhancement, without directly targeting vascular or endothelial pathobiology.

Other recently developed or emerging therapies, including selective cardiac myosin inhibitors/activators and agents modulating inflammation or iron deficiency, address distinct dimensions of heart failure vulnerability but do not directly correct the NO–sGC–cGMP axis. Accordingly, vericiguat may be best understood as a pathway-specific adjunct for carefully selected high-risk patients with recent decompensation despite guideline-directed therapy, whereas ARNI and SGLT2 inhibitors remain foundational disease-modifying therapies across a broader spectrum, and omecamtiv mecarbil occupies a more phenotype-driven role in patients with persistent systolic dysfunction. This framing supports a layered treatment model in which therapy selection is guided not only by ejection fraction and symptoms, but also by timing after destabilization, dominant residual pathophysiology, and the specific mechanism each drug class is designed to address.

A comparison with other contemporary and emerging HFrEF therapies underscores that vericiguat occupies a distinct mechanistic position: unlike sacubitril/valsacor (ARNI), sodium-glucose transmembrane transporter inhibitor-2 (SGLT2i), omecamtiv mecabril, ivabradine, intravenous iron which have broader foundational roles across the heart failure continuum, or omecamtiv mecarbil, which primarily augments systolic performance, vericiguat is uniquely aligned with the correction of residual NO–sGC–cGMP pathway dysfunction in the early post-stabilization period. To improve comparison across therapeutic classes, Table 4 focuses on the principal mechanisms and clinical roles most relevant to residual-risk targeting in HFrEF.

Moreover, the recent evidence suggests that the benefits of contemporary HF therapies extend beyond traditional hemodynamic effects and include modulation of inflammation, endothelial function, myocardial energetics, and cardiorenal interactions [55]. These observations reinforce the concept that residual biological risk may persist despite clinical stabilization and support ongoing investigation into complementary pathway-specific therapies.

## 8. Future Directions

Future research should focus on defining both the biological substrate and the temporal window in which soluble guanylate cyclase stimulation provides the greatest clinical benefit.

Five specific priorities emerge from the current evidence base. First, trials should evaluate vericiguat initiation during the early post-stabilization window after worsening HF. Second, the incremental value of vericiguat should be tested on top of fully optimized contemporary quadruple therapy. Third, dedicated studies are needed in patients with ACS-triggered worsening HFrEF or persistent LV dysfunction after myocardial infarction. Fourth, biomarker-guided strategies incorporating NT-proBNP, inflammatory markers, and endothelial function indices should be developed to refine patient selection. Fifth, the potential anti-arrhythmic and sudden-death–modifying effects suggested by recent data require mechanistic and prospective validation.

Although current evidence supports the use of vericiguat in patients with recently worsening HFrEF, important questions remain regarding patient selection, timing of initiation, and integration with contemporary heart failure therapy.

A major priority is the prospective evaluation of patients during the early post-stabilization period following worsening heart failure. Future trials should specifically enroll patients within the first weeks after hospitalization or intravenous diuretic-treated decompensation, a phase characterized by persistently elevated residual risk despite apparent clinical recovery. Such studies should assess whether the addition of vericiguat to fully optimized contemporary quadruple therapy provides incremental benefit beyond current standards of care.

An equally important objective is the identification of biological markers capable of refining patient selection. Persistent endothelial dysfunction, impaired NO–sGC–cGMP signaling, and ongoing vascular inflammation represent attractive mechanistic targets, yet no validated clinical strategy currently exists to identify these processes in routine practice. Future investigations should therefore evaluate biomarker-guided approaches incorporating natriuretic peptides, inflammatory markers, indices of endothelial function, and emerging molecular signatures to determine whether specific biological phenotypes derive greater benefit from sGC stimulation.

The potential role of vericiguat across the broader ischemia–decompensation continuum also warrants further investigation. In particular, patients who develop left ventricular dysfunction following acute coronary syndromes and subsequently enter a period of clinical stabilization represent a biologically plausible but largely unstudied population. Dedicated prospective studies will be necessary to determine whether the mechanistic rationale observed in worsening HFrEF can be translated into a clinically meaningful benefit in selected post-ACS patients.

Future studies should additionally evaluate the position of vericiguat within modern multidrug treatment strategies. Because the VICTORIA trial preceded widespread implementation of contemporary quadruple therapy, the incremental value of vericiguat in patients receiving optimized ARNI, beta-blockers, mineralocorticoid receptor antagonists, and SGLT2 inhibitors remains incompletely defined. Clarifying its additive benefit within current treatment paradigms will be essential for determining its optimal clinical niche.

More broadly, future research may benefit from moving beyond traditional disease-based classifications toward mechanism-based and trajectory-based trial designs. Rather than focusing exclusively on diagnostic categories, studies should consider the dynamic evolution of cardiovascular disease and target patients according to biological vulnerability, residual pathophysiological activation, and timing after destabilization. Such an approach may improve therapeutic precision and facilitate the development of personalized strategies aimed at reducing residual risk during the vulnerable post-stabilization phase.

To facilitate interpretation of the available data and to distinguish between established clinical knowledge and areas requiring further investigation, Table 5 summarizes the current evidence base for vericiguat in HFrEF. The table categorizes findings into three domains: established evidence derived from randomized clinical trials, emerging concepts supported by mechanistic and translational research, and unresolved questions that remain the focus of future studies. This framework highlights where vericiguat’s clinical role is well defined—particularly in patients with recently worsening HFrEF—and where additional evidence is needed, including the optimal timing of initiation, biomarker-guided patient selection, integration with contemporary quadruple therapy, potential application in post-ACS populations, and the significance of possible anti-arrhythmic effects. By presenting these aspects together, the table provides a concise overview of the current state of knowledge and helps identify priorities for future research and clinical development.

## 9. Translational Perspective

From a translational perspective, the proposed continuum-based framework emphasizes aligning therapeutic strategies with dynamic disease states rather than static diagnostic categories. Biomarker-guided identification of patients with persistent endothelial dysfunction or impaired cGMP signaling may further refine this approach. Ultimately, integrating mechanistic insights with temporal disease profiling could enable more precise and individualized use of therapies such as vericiguat.

### Limitations

Several limitations should be acknowledged. The magnitude of benefit observed with vericiguat is modest, and its efficacy appears to be dependent on patient selection, particularly with respect to baseline risk and natriuretic peptide levels. In addition, data in populations outside the VICTORIA inclusion criteria, including patients early after ACS without established HFrEF, remain limited. Finally, the optimal timing of initiation and integration with contemporary multi-drug regimens requires further clarification. These uncertainties highlight the need for prospective studies specifically targeting the early post-stabilization period.

Furthermore, the conceptual framework proposed in this manuscript has not been prospectively tested and should therefore be interpreted as a hypothesis intended to inform future research rather than to guide immediate changes in clinical practice.

Despite the growing body of evidence supporting vericiguat in patients with recently worsening HFrEF, several important limitations and knowledge gaps remain. The current evidence base is largely derived from a specific high-risk population enrolled in the VICTORIA trial and may not be fully generalizable to contemporary patients receiving optimized quadruple guideline-directed medical therapy. Furthermore, evidence in populations outside the established indication, particularly patients following acute coronary syndromes without worsening heart failure, remains limited and largely exploratory. Uncertainties also persist regarding the optimal timing of initiation, biomarker-guided patient selection, and the potential clinical relevance of proposed anti-arrhythmic effects. Table 6 summarizes the principal limitations of the current evidence base and highlights key areas requiring prospective investigation before broader implementation of vericiguat can be considered.

## 10. Conclusions

Vericiguat represents a novel therapeutic approach targeting the NO–sGC–cGMP pathway in patients with HFrEF. While currently indicated for those with recent worsening, its greatest value may lie within a broader clinical context.

Vericiguat represents a mechanistically distinct therapeutic option targeting the NO–sGC–cGMP pathway in patients with HFrEF. While current evidence supports its use in patients with recent worsening, its potential role within the early post-stabilization phase remains hypothesis driven. Importantly, the proposed ischemia–decompensation continuum should not be interpreted as evidence supporting vericiguat across all phases of ischemic heart disease. Rather, it serves as a conceptual framework to identify biological vulnerability within the worsening HFrEF population and to generate hypotheses for future investigation.

Viewing cardiovascular disease as a dynamic continuum—from ischemia through decompensation to chronic heart failure—may help identify a period of heightened vulnerability in which targeted intervention could be particularly relevant. However, prospective studies specifically designed to address this transitional phase are required to determine whether such an approach can meaningfully reduce residual risk beyond contemporary therapy.

## Figures and Tables

**Table 1:** Pathophysiological derangements in post-stabilization state.

Feature	Pathophysiological Basis	Potential Clinical Assessment	Why Vericiguat May Be Relevant?	Key References
Residual endothelial dysfunction	Reduced NO bioavailability, oxidative stress, impaired vasodilatory response, endothelial activation	hsCRP, NT-proBNP, flow-mediated dilation (FMD), peripheral arterial tonometry (PAT), coronary microvascular function assessment	Direct sGC stimulation restores downstream cGMP signaling despite reduced NO availability	[15,16,26,27,28]
Persistent neurohormonal activation	Continued activation of RAAS and sympathetic nervous system despite clinical stabilization	NT-proBNP, heart rate, blood pressure, congestion assessment	Complements neurohormonal blockade by targeting a distinct pathway (NO–sGC–cGMP)	[3,7,29,30]
Inflammatory activation	Elevated cytokines, NF-κB signaling, persistent low-grade inflammation	hsCRP, IL-6, inflammatory biomarkers	cGMP signaling may attenuate inflammatory and oxidative pathways	[14,15,26,28]
cGMP deficiency	Reduced NO signaling and impaired sGC activation leading to diminished intracellular cGMP	No routine clinical test available; inferred from endothelial dysfunction and disease severity	Vericiguat directly stimulates sGC and restores cGMP production	[25,28,31,32]
Microvascular dysfunction/vascular stiffness	Impaired endothelial regulation, increased afterload, reduced vascular compliance	Pulse-wave velocity, coronary flow reserve, echocardiographic and vascular assessments	Increased cGMP-PKG signaling promotes vasodilation and improves vascular function	[16,25,26,31]
Incomplete myocardial recovery	Persistent myocardial injury, fibrosis, adverse remodeling	Echocardiography, cardiac MRI, NT-proBNP	Improved cGMP signaling may reduce myocardial stress and maladaptive remodeling	[17,18,33]
Arrhythmic substrate	Electrical instability, CaMKII activation, altered calcium handling	ECG, Holter monitoring, device interrogation	Experimental data suggest anti-arrhythmic effects through modulation of intracellular signaling	[33,34]
Elevated residual clinical risk	Combined effect of persistent biological activation despite apparent stabilization	Recent HF hospitalization, IV diuretic requirement, elevated NT-proBNP, recurrent HF events	Matches the clinical profile in which vericiguat demonstrated benefit in VICTORIA	[23,24,35,36]

Abbreviations: CaMKII—Calcium/Calmodulin-Dependent Protein Kinase II, cGMP—Cyclic Guanosine Monophosphate, NF-κB—Nuclear Factor Kappa B, NO—Nitric Oxide, PKG—Protein Kinase G, sGC—Soluble Guanylate Cyclase, VT—Ventricular Tachycardia.

**Table 2:** Victoria and Victor’s trials key findings.

Trial	Population	Primary Endpoint	Key Finding	Key References
VICTORIA (2020)	Worsening HF (hospitalization or IV diuretics within 6 months)	CV death or HF hospitalization	Positive (HR 0.90, *p* = 0.02)	[35,40]
VICTOR (2025)	Stable HFrEF, no recent worsening	CV death or HF hospitalization	Neutral (HR 0.93, *p* = NS)	[36,41]

Abbreviations: CV—cardiovascular, HF—heart failure, HFrEF—heart failure with reduced ejection fraction, HR—hazard ratio, IV—intravenous, NS—non-significant.

**Table 3:** Timing-based positioning of vericiguat across the heart failure trajectory. Evidence supporting vericiguat is strongest in patients with worsening HFrEF after clinical stabilization, consistent with the VICTORIA population. Application to post-ACS populations without worsening HFrEF remains exploratory and should be regarded as hypothesis-generating.

Clinical Phase	Patient Profile	Evidence for Vericiguat	Potential Clinical Role	Recommendation Level	Key References
Acute decompensation (during hospitalization)	Hemodynamic instability, ongoing IV diuretics/inotropes, SBP < 100 mmHg	No randomized evidence supporting initiation during acute instability	Safety and efficacy not established	Not recommended	[35,44]
Early stabilization (before discharge or shortly after discharge)	Congestion resolved, no IV therapy ≥ 24 h, SBP ≥ 100 mmHg	Consistent with population entering VICTORIA after stabilization	Potential initiation window in eligible patients with worsening HFrEF	Supported by available evidence	[35,36,44]
Post-stabilization vulnerable phase (approximately 7–90 days after worsening HF event)	Recent HF hospitalization or IV diuretic-treated worsening HF, elevated NT-proBNP, persistent residual risk despite GDMT	Strongest evidence base from VICTORIA	Reduction in composite of CV death or HF hospitalization, primarily driven by fewer HF events	Preferred clinical setting	[34,35,36,41,42,44]
Chronic stable HFrEF (>90 days without recent worsening)	Clinically stable patients receiving optimized GDMT	Benefit less consistent; VICTOR findings differ from VICTORIA population	Individualized consideration in selected high-risk patients	Evidence limited	[34,41,42]
Post-ACS without worsening HFrEF	Recent ACS with reduced EF but no HF worsening event	Evidence exploratory and limited	Mechanistic rationale only; requires prospective validation	Hypothesis-generating	[43]

Abbreviations: ACS, acute coronary syndrome; CV, cardiovascular; EF, ejection fraction; GDMT, guideline-directed medical therapy; HF, heart failure; HFrEF, heart failure with reduced ejection fraction; IV, intravenous; NT-proBNP, N-terminal pro-B-type natriuretic peptide; SBP, systolic blood pressure; VICTOR, Vericiguat Global Study in Participants with Chronic Heart Failure; VICTORIA, Vericiguat Global Study in Subjects with Heart Failure with Reduced Ejection Fraction.

**Table 4:** HFrEF therapies.

Therapy/Class	Main Mechanism	Recommended Clinical Setting	Principal Clinical Benefit	Relationship to Vericiguat	Key References
ARNI (sacubitril/valsartan)	Enhances natriuretic peptide signaling and suppresses RAAS activation	Foundational therapy for symptomatic HFrEF	Reduces CV mortality and HF hospitalization	Remains first-line therapy; vericiguat should be considered only after optimization of foundational therapy	[3,30,47]
β-blockers	Inhibit sympathetic activation	Foundational therapy for all eligible HFrEF patients	Improves survival and reduces hospitalization	Essential background therapy before considering vericiguat	[3,30,47]
MRAs	Block mineralocorticoid receptor-mediated remodeling and fibrosis	Foundational therapy for HFrEF	Reduces mortality and HF hospitalization	Complementary mechanism; should be optimized before adding vericiguat	[3,30,47]
SGLT2 inhibitors	Cardiorenal and metabolic effects; reduction in congestion and inflammation	Foundational therapy across the HFrEF spectrum	Rapid reduction in HF hospitalization and CV events	Complementary therapy; not directed at NO–sGC–cGMP signaling	[29,30,47,48,49,50]
Vericiguat	Direct stimulation of soluble guanylate cyclase with restoration of cGMP signaling	HFrEF with recent worsening despite GDMT	Reduction in composite of CV death or HF hospitalization, primarily through fewer HF events	Adjunctive therapy targeting residual risk and impaired NO–sGC–cGMP signaling	[34,35,36,41,42]
Ivabradine	Heart-rate reduction via If-channel inhibition	Sinus rhythm with HR ≥ 70 bpm despite maximally tolerated β-blocker	Reduces HF hospitalization	Phenotype-specific therapy; complementary to vericiguat	[47]
Intravenous iron	Correction of iron deficiency and impaired cellular energetics	HFrEF with iron deficiency	Improves symptoms, exercise capacity, and may reduce recurrent HF events	Addresses metabolic vulnerability rather than endothelial dysfunction	[47,48]
Omecamtiv mecarbil	Selective cardiac myosin activation	Selected patients with severe systolic dysfunction	Modest reduction in HF events	Targets contractility rather than vascular dysfunction	[30]

Abbreviations: AFIRM-AHF—A randomized, double-blind trial of intravenous iron in acute heart failure, ARNI—Angiotensin Receptor–Neprilysin Inhibitor, BP—Blood Pressure, cGMP—Cyclic Guanosine Monophosphate, CV—Cardiovascular, DA-PA-HF—Dapagliflozin and Prevention of Adverse Outcomes in Heart Failure trial, EF—Ejection Fraction, EMPEROR-Reduced—Empagliflozin Outcome Trial in Patients with Chronic Heart Failure with Reduced Ejection Fraction, EMPULSE—Empagliflozin in Patients Hospitalized for Acute Heart Failure, GALACTIC-HF—Global Approach to Lowering Adverse Cardiac Outcomes Through Improving Contractility in Heart Failure, HF—Heart Failure, HEART-FID—Heart Failure with Iron Deficiency trial, HFrEF—Heart Failure with Reduced Ejection Fraction, HR—Heart Rate, IRONMAN—Intravenous Iron Treatment in Patients with Heart Failure and Iron Deficiency, IV—Intravenous, NO—Nitric Oxide, PARADIGM-HF—Prospective Comparison of ARNI with ACEI to Determine Impact on Global Mortality and Morbidity in Heart Failure, PIONEER-HF—Comparison of Sacubitril–Valsartan vs. Enalapril on Effect on NT-proBNP in Patients Stabilized from an Acute Heart Failure Episode, RAAS—Renin–Angiotensin–Aldosterone System, sGC—Soluble Guanylate Cyclase, SHIFT—Systolic Heart Failure Treatment with the If Inhibitor Ivabradine Trial, SGLT2—Sodium–Glucose Cotransporter 2, SOLOIST-WHF—Sotagliflozin in Patients with Diabetes and Recent Worsening Heart Failure, TRANSITION—Initiation of Sacubitril/Valsartan in Heart Failure Patients Stabilized After Acute Decompensation, VICTORIA—Vericiguat Global Study in Subjects with Heart Failure with Reduced Ejection Fraction.

**Table 5:** Established evidence, emerging concepts, and unresolved questions for vericiguat in HFrEF.

Established Evidence	Emerging Concept	Unresolved Question
Vericiguat reduces CV death or HF hospitalization in worsening HFrEF	Benefit may be greatest during early post-stabilization vulnerability	What is the optimal timing after stabilization?
Vericiguat targets the NO–sGC–cGMP pathway	Residual endothelial dysfunction may identify higher-risk patients	Can biomarkers guide patient selection?
Current guidelines position vericiguat as add-on therapy after worsening HF	It may complement quadruple therapy mechanistically	What is the incremental value on top of ARNI, BB, MRA, and SGLT2i?
Post-ACS use remains unsupported by direct evidence	ACS-triggered worsening HFrEF may be a biologically plausible subgroup	Should post-ACS HFrEF be tested in dedicated trials?
Mortality/SCD signals remain exploratory	Anti-arrhythmic effects are biologically plausible	Are these effects clinically meaningful and reproducible?

Abbreviations: ACS—Acute Coronary Syndrome; ARNI—Angiotensin Receptor–Neprilysin Inhibitor; BB—Beta-Blocker; CV—Cardiovascular; GDMT—Guideline-Directed Medical Therapy; HF—Heart Failure; HFrEF—Heart Failure with Reduced Ejection Fraction; MRA—Mineralocorticoid Receptor Antagonist; NO—Nitric Oxide; sGC—Soluble Guanylate Cyclase; cGMP—Cyclic Guanosine Monophosphate; SCD—Sudden Cardiac Death; SGLT2i—Sodium–Glucose Cotransporter 2 Inhibitor.

**Table 6:** Key limitations of the current evidence base.

Limitation	Implication
VICTORIA was conducted before widespread use of SGLT2 inhibitors and contemporary quadruple therapy	Incremental benefit in modern GDMT-treated patients remains uncertain
Post-ACS evidence is limited and exploratory	Routine use after ACS without worsening HFrEF cannot be recommended
No validated endothelial dysfunction-based selection algorithm exists	Biomarker-guided use remains investigational
Effect size in VICTORIA was modest and mainly driven by HF hospitalization	Expectations regarding mortality benefit should remain cautious
Optimal timing after stabilization is not definitively established	Prospective timing-based trials are needed
Anti-arrhythmic/SCD effects remain hypothesis-generating	Dedicated mechanistic studies are required

Abbreviations: ACS, acute coronary syndrome; GDMT, guideline-directed medical therapy; HF, heart failure; HFrEF, heart failure with reduced ejection fraction; SCD, sudden cardiac death; SGLT2, sodium–glucose cotransporter 2; VICTORIA, Vericiguat Global Study in Subjects with Heart Failure with Reduced Ejection Fraction.

## Data Availability

No data were created for the study.

## Abbreviations

The following abbreviations are used in this manuscript:

ACS	Acute Coronary Syndrome
AFIRM-AHF	A Randomized, Double-Blind Trial of Intravenous Iron in Acute Heart Failure
AHF	Acute Heart Failure
APD	Action Potential Duration
ARNI	Angiotensin Receptor–Neprilysin Inhibitor
BB	Beta-Blocker
BP	Blood Pressure
CaMKII	Calcium/Calmodulin-Dependent Protein Kinase II
cGMP	Cyclic Guanosine Monophosphate
CV	Cardiovascular
EF	Ejection Fraction
EMPEROR-Reduced	Empagliflozin Outcome Trial in Patients with Chronic Heart Failure with Reduced Ejection Fraction
EMPULSE	Empagliflozin in Patients Hospitalized for Acute Heart Failure
eGFR	Estimated Glomerular Filtration Rate
GALACTIC-HF	Global Approach to Lowering Adverse Cardiac Outcomes Through Improving Contractility in Heart Failure
GDMT	Guideline-Directed Medical Therapy
HF	Heart Failure
HEART-FID	Heart Failure with Iron Deficiency Trial
HFrEF	Heart Failure with Reduced Ejection Fraction
HR	Hazard Ratio/Heart Rate (define per context if required by journal)
IRONMAN	Intravenous Iron Treatment in Patients with Heart Failure and Iron Deficiency
IV	Intravenous
MRA	Mineralocorticoid Receptor Antagonist
NF-κB	Nuclear Factor Kappa B
NO	Nitric Oxide
NT-proBNP	N-terminal pro–B-type Natriuretic Peptide
PARADIGM-HF	Prospective Comparison of ARNI with ACEI to Determine Impact on Global Mortality and Morbidity in Heart Failure
PCI	Percutaneous Coronary Intervention
PIONEER-HF	Comparison of Sacubitril–Valsartan vs. Enalapril on Effect on NT-proBNP in Patients Stabilized from an Acute Heart Failure Episode
PKG	Protein Kinase G
RAAS	Renin–Angiotensin–Aldosterone System
SBP	Systolic Blood Pressure
SCD	Sudden Cardiac Death
sGC	Soluble Guanylate Cyclase
SHIFT	Systolic Heart Failure Treatment with the If Inhibitor Ivabradine Trial
SGLT2	Sodium–Glucose Cotransporter 2
SGLT2i	Sodium–Glucose Cotransporter 2 Inhibitor
SOLOIST-WHF	Sotagliflozin in Patients with Diabetes and Recent Worsening Heart Failure
TRANSITION	Initiation of Sacubitril/Valsartan in Heart Failure Patients Stabilized After Acute Decompensation
VICTOR	Vericiguat Global Study in Participants with Chronic Heart Failure
VICTORIA	Vericiguat Global Study in Subjects with Heart Failure with Reduced Ejection Fraction
VT	Ventricular Tachycardia
